# Histone deacetylase inhibitor trichostatin A sensitises cisplatin-resistant ovarian cancer cells to oncolytic adenovirus

**DOI:** 10.18632/oncotarget.25242

**Published:** 2018-05-29

**Authors:** Sarah L. Hulin-Curtis, James A. Davies, Rachel Jones, Emma Hudson, Louise Hanna, John D. Chester, Alan L. Parker

**Affiliations:** ^1^ Division of Cancer and Genetics, School of Medicine, Cardiff University, Heath Park, Cardiff, CF14 4XN, UK; ^2^ Velindre Cancer Centre, Whitchurch, Cardiff, CF14, 2TL, UK

**Keywords:** histone deacetylase inhibitor, oncolytic adenovirus, cisplatin-resistance, ovarian cancer

## Abstract

Ovarian cancer is often termed a silent killer due to the late onset of symptoms. Whilst patients initially respond to chemotherapy, they rapidly develop chemo-resistance. Oncolytic adenoviruses (OAds) are promising anti-cancer agents engineered to “hijack” the unique molecular machinery of cancer cells enabling tumour-selective viral replication. This allows spread to adjacent cells and amplification of oncolysis within the tumour. OAds represent an excellent opportunity for ovarian cancer therapy via intra-peritoneal delivery, however the efficacy of OAds thus far is limited. Here, we evaluate chromatin (histone) modification in chemo-resistant cells and its relationship to Ad efficacy (wild-type or oncolytic Ad). In contrast to cisplatin-sensitive A2780 cells that show an efficient reduction of cell viability by Ad in the presence of cisplatin, cisplatin-resistant A2780/cp70 cells show diminishing Ad-mediated reduction of cell viability with escalating doses of cisplatin. Histone deacetylase (HDAC)-2 and to a lesser extent HDAC1 were up-regulated in cisplatin-resistant but not cisplatin-sensitive cells. Cisplatin-resistant cells treated with a pan-HDAC inhibitor trichostatin A (TsA) significantly enhanced Ad-mediated reduction of cell viability in the presence of cisplatin. Cells treated with TsA alone did not reduce cell viability suggesting these findings are Ad-dependent. Thus, we identify HDAC inhibition as a potential means to sensitise cisplatin-resistant ovarian cancer cells to virotherapies, an observation that may offer improved outcomes for patients with late stage, chemotherapy-resistant ovarian cancer.

## INTRODUCTION

Worldwide, nearly 239,000 women were estimated to have been diagnosed with ovarian cancer and around 65,600 new cases diagnosed in Europe in 2012 [1]. Patients commonly present at an advanced, often incurable stage, because of the late onset of symptoms. Although around 80% of patients’ tumours respond initially to cytotoxic chemotherapy (usually up to two years), nearly all patients ultimately develop chemo-resistance and relapse [2]. Early observations of reduced tumour growth in response to vaccination prompted efforts to develop treatments based on biologics such as Ads [3]. Since then, OAds have been widely developed as therapies for cancer treatment [4]. There are 57 human Ad serotypes but those based on the species C Ad5 have been most widely evaluated for virotherapy applications. Ad5 can be readily manipulated by genetic or chemical means and amplified to high titers. However, several challenges limit the efficacy of Ad5 clinically. Infection is dependent on cell entry via the native receptor, coxsackie and adenovirus receptor (CAR) [5]. CAR expression is frequently down-regulated as a function of tumour progression [6–8]. Therefore, efforts to target alternative receptors are under investigation [9–11]. Ad5 is an endemic virus causing upper respiratory tract infections with up to 90% of the population having developed neutralising antibodies (nAbs) against Ad5 [12, 13]. Consequently, pre-existing nAbs results in rapid, efficient elimination of Ad5 vectors upon *in vivo* systemic delivery [14, 15]. Ascites, an accumulation of fluid within the patient’s abdomen, represents a hallmark feature of ovarian cancer and contains nAbs as well as multiple cell types influencing the tumour microenvironment and response to chemotherapy [16, 17].

Clinical studies of OAds have been met with mixed success [18]. Onyx-015 and H101 (Oncorine) used in combination with chemotherapy agents in head and neck cancer showed little cell killing activity *in vivo* [19–23]. Imlygic (talimogene laherparepvec or T-Vec) developed for malignant melanoma [24] showed varying patient response at Phase III trial [25]. Efforts to develop more efficacious cancer immunotherapies by combining oncolytic virotherapy with chemotherapy, immune checkpoint modulators and epigenetic modifiers is attracting renewed interest [26–28]. Almost without exception, cancer cells display epigenetic (histone) aberrations that are linked to the development and progression of cancer [18, 29] and chemo-resistance [30]. HDAC inhibitors (HDACis) block histone deacetylation, promote cancer cell death and an immunogenic response [31]. These include vorinostat, romidepsin (FR901228, depsipeptide) and belinostat which have gained approval for haematological and solid malignancies [18].

It is well accepted that in addition to disturbances of histone acetyltransferase (HAT) and histone deacetyltransferase (HDAC) activity in tumour development, both enzymes are able to target non-histone targets such as viruses and proteins involved in cellular proliferation, migration, apoptosis and DNA repair [32]. The lack of efficacy of OAds in clinical trials prompted us to evaluate in this study whether potential differences in histone status between chemo-sensitive and-resistant ovarian cancer cells affect OAd efficacy. We hypothesised that Ad infection and replication hence efficacy might be altered with differences in histone regulation between these cell types. In the present *in vitro* study, we developed a control Ad (replication-deficient), replication-competent Ad5 wild-type (Ad5WT) and conditionally-replicating dl24 (∆24) rendered oncolytic by deletion of 24 base-pairs (amino acids 120-127) in the adenoviral *E1A* region [33]. We compared the efficacy of Ads in cisplatin-sensitive and matched -resistant ovarian cancer cells. Our findings outline a novel potential role for histone deacetylation inhibition in improving OAd-mediated reduction of cell viability of platinum-resistant ovarian cancer cells. HDACis may have important clinical implications for future combination trials in end-stage ovarian cancer patients.

## RESULTS

### Characterisation of the CAR receptor shows heterogeneity between patient EOC cells and similar expression in ovarian cancer cell lines

The native Ad5 receptor CAR is required for Ad infection [5]. In order to characterise the efficacy of our panel of Ads for this study, we sought to first determine the expression levels of CAR on primary epithelial ovarian cancer (EOC) cells cultured from clinical ascites [11] (data not shown). The composition of cells derived from metastatic sites of ovarian cancer patients in ascites varies widely and comprises tumour, mesothelial, fibroblast, immune and red blood cells. We selected two samples with contrasting CAR levels to test whether significantly different expression levels of CAR influenced Ad efficacy in our chemo-resistance model. EOC003 primary tumour cells, donated by a patient with end-stage, chemo-resistant disease, showed 40% of cells in total were positive for CAR expression as determined by flow cytometry whilst EOC009 cells, derived from a patient with chemo-sensitive disease expressed 99% CAR. A2780 cisplatin-sensitive and A2780/cp70 cisplatin-resistant cells expressed approximately 30% and 36% CAR expression, respectively (data not shown).

### dl24 efficacy is comparable to Ad5WT

**Figure 1:**
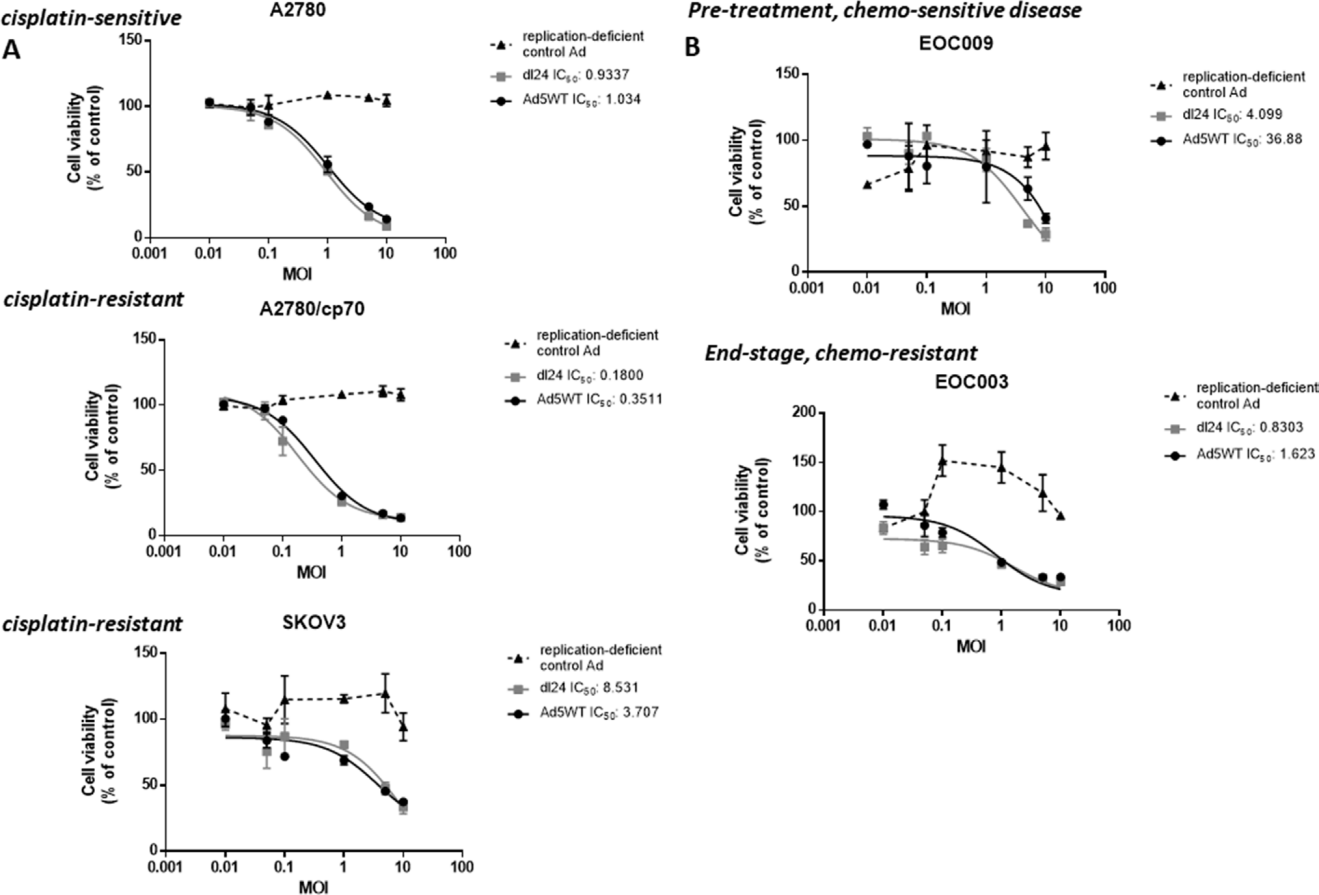
Ad5WT (wild-type replication-competent) and dl24 (oncolytic) Ad show specific and dose-dependent reductions in cell viability in (**A**) ovarian cancer cell lines and (**B**) primary epithelial ovarian cancer (EOC) cells derived from patient ascitic fluid. No reduction of cell viability was observed for the control Ad (replication-deficient). A2780 (cisplatin-sensitive) and A2780/cp70 (cisplatin-resistant), SKOV3 and primary EOC (EOC003 and EOC009) cell viability in response to Ad infection (0–10 MOI) was quantified by MTS assay at 72 h post-infection. Cell viability was calculated as a percentage of uninfected cells (control). Data was corrected for background absorbance from the incubation media. Data is expressed as the mean ± SEM. Experiments were performed in triplicate.

We next tested the efficacy of our panel of Ads by performing a dose-response experiment, infecting cells at a multiplicity of infection (MOI) of 0-10 in matched cisplatin-sensitive and cisplatin-resistant ovarian cancer cell lines (A2780 and A2780/cp70 respectively) and primary epithelial ovarian cancer (EOC) tumour cell cultures (Figure 1). The replication-deficient control Ad did not reduce cell viability in any cell type. There was no statistically significant difference in cell viability for any cell type infected with Ad5WT except in SKOV3 cells where Ad5WT decreased cell viability (*P <* 0.05). We observed a significant decrease in cell viability for A2780, SKOV3 and EOC003 cells (end-stage, chemo-resistant disease) infected with dl24 in comparison to the replication-deficient control Ad (*P* < 0.05).

### Cisplatin reduces Ad efficacy in cisplatin-resistant cells

**Figure 2:**
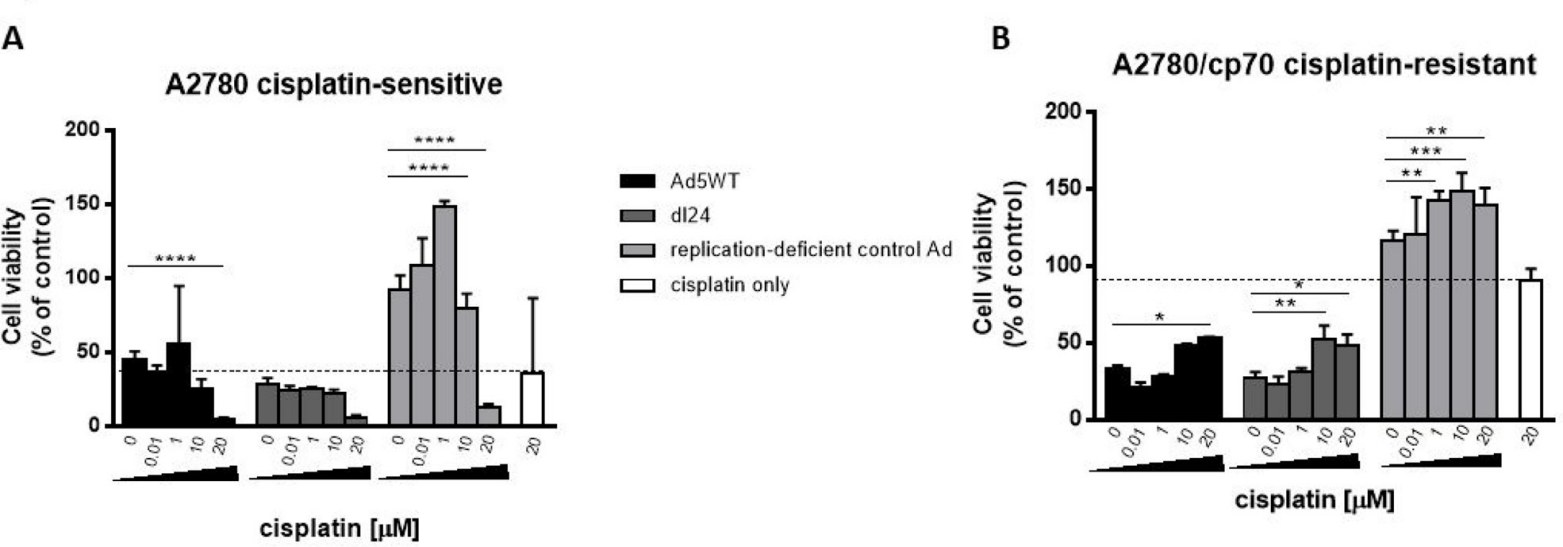
(**A**) A2780 (cisplatin-sensitive) cells show enhanced Ad-mediated reduction of cell viability with escalating dose of cisplatin, whereas (**B**) A2780/cp70 (cisplatin-resistant) cells show reduced Ad-mediated reduction in cell viability with escalating dose of cisplatin. Cells were infected with Ad5WT (replication-competent), dl24 (oncolytic) and control Ad (replication-deficient) at an MOI of 10 and treated with escalating doses of cisplatin (0–20 µM). Cell viability was measured by MTS assay at 72 h post-infection. Cell viability was calculated as a percentage of uninfected cells (control). Data was corrected for background absorbance from the incubation media. Data is expressed as the mean ± SEM. Experiments were performed in triplicate.

A major clinical limitation for the treatment of ovarian cancer is the rapid development of chemo-resistance. We therefore tested the efficacy of our Ads, as an alternative or adjunct to cisplatin, in cisplatin-sensitive and matched -resistant cells (Figure 2). Both wild-type replication-competent Ad5 (Ad5WT) and selectively replicating dl24 showed cytotoxic effects that were similar to or better than 20 µM cisplatin, alone or in combination with cisplatin, in cisplatin-sensitive A2780 cells. In matched cisplatin-resistant A2780/cp70 cells, both Ads were much more effective than cisplatin when administered as a single agent and, in contrast to the cisplatin dose-effect seen in the A2780 cells, efficacy was reduced by co-administration of increasing concentrations of cisplatin.

### Ad transduction is comparable in cisplatin-sensitive and -resistant cells

To rule out any bias in our observed differences in Ad-mediated reduction in cell viability between cisplatin-sensitive and -resistant cells, we performed transduction experiments by infecting both cell types with dl24 at 1000, 5000 and 10,000 virus particles (vp)/cell. We observed no difference in transduction activity between cisplatin-sensitive or -resistant cells, as assessed by expression of a virally-encoded transgene (Supplementary Figure 1).

### HDAC2 is up-regulated in cisplatin-resistant cells but HDAC2 knockdown has no effect on cell viability

**Figure 3:**
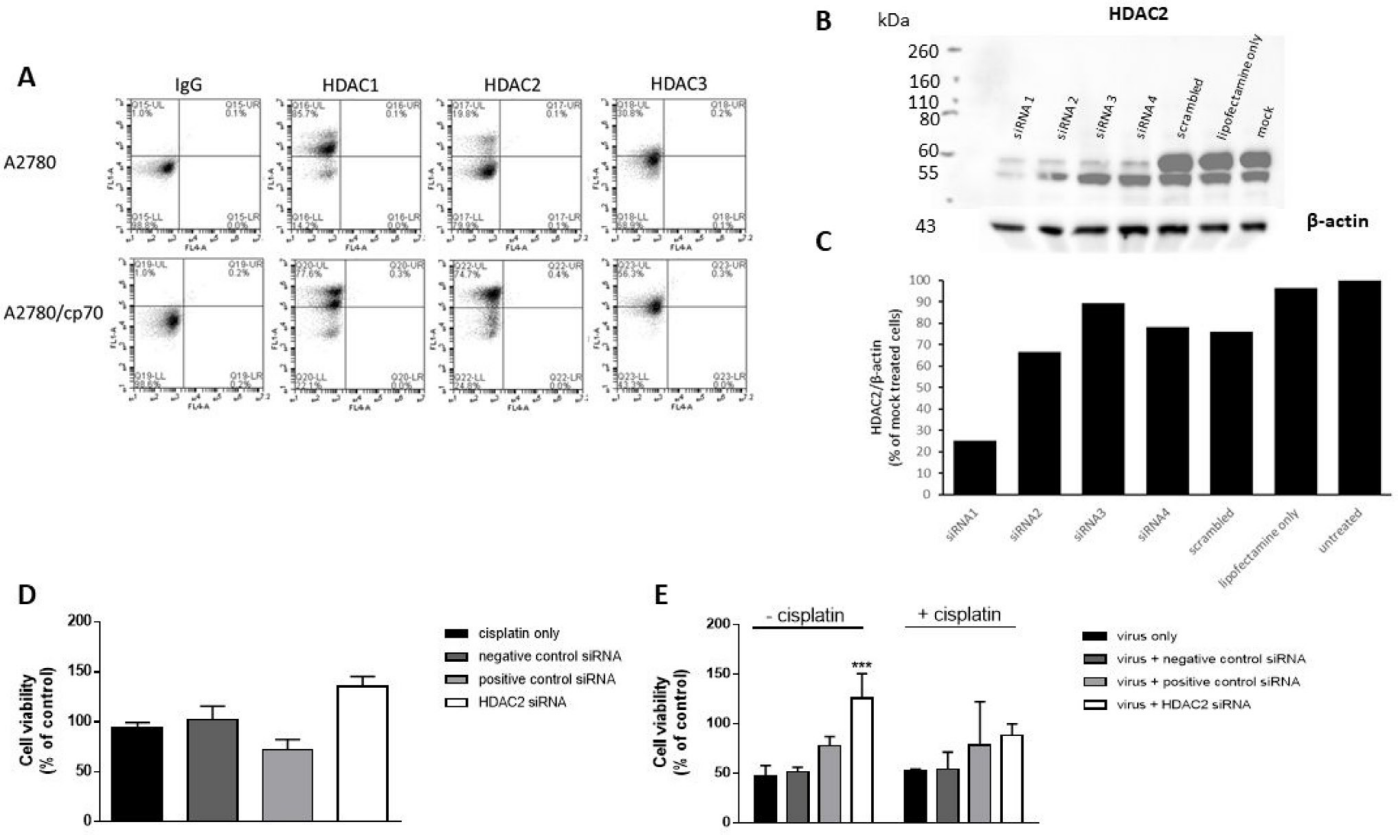
HDAC2 expression is enhanced in A2780/cp70 (cisplatin-resistant) cells in comparison to A2780 (cisplatin-sensitive cells) but knockdown of HDAC2 has no effect on cisplatin-resistant cell proliferation (**A**) Flow cytometry data shows quantification of HDAC1, HDAC2 and HDAC3 expression in both cell types. (**B**) Representative Western Blot to show HDAC2 protein levels after siRNA 1, 2, 3 and 4 knockdown and β-actin loading control in A2780/cp70 cisplatin-resistant cells. (**C**) Corresponding densitometry plots from (B). (**D**) Cell viability (% of uninfected control) of A2780/cp70 cells treated with cisplatin, negative control (scramble) siRNA, positive control (AbI 1) siRNA or HDAC2 (siRNA1) siRNA. (**E**) Cell viability (% of uninfected control) of A2780/cp70 cells infected with dl24 oncolytic Ad in the presence and absence of cisplatin after HDAC2 knockdown by siRNA (siRNA1). Experiments were performed in triplicate. Data is expressed as the mean ± SEM. Statistics were performed using the Sidak`s multiple comparison test. *P* values ≤ 0.05 are considered statistically significant and calculated to determine differences between Ad infected cells with and without different siRNAs.

The emergence of chemo-resistance has been associated with epigenetic modifications [30]. We sought to determine whether epigenetic histone modifications might be responsible for the observed differences in Ad efficacy in cisplatin-treated A2780 and A2780/cp70 cells. We examined the expression profiles of nuclear HDAC1, HDAC2 and HDAC3 by flow cytometry (Figure 3A). A2780/cp70 cisplatin-resistant cells showed a marked increase in HDAC2 expression in comparison to cisplatin-sensitive A2780 cells (from 74.7% to 19.8% respectively). We therefore sought to determine whether knockdown of HDAC2 by siRNA reverses the chemo-resistant phenotype. We achieved knockdown of HDAC2 using siRNA 1 (Figure 3B) in A2780/cp70 cells and quantified knockdown of 76% as measured by densitometry (Figure 3C). However HDAC2 knockdown failed to significantly alter cell viability (Figure 3D) suggesting HDAC2 alone is not responsible for the chemo-resistant phenotype of A2780/cp70 cells. HDAC1 and HDAC2 share 83% sequence homology and exist together as a co-repressor complex. Knockdown of either HDAC1 or HDAC2 in liver and colorectal cancer cell lines has little effect on cell viability and proliferation, whereas combined knockdown of HDAC1 and HDAC2 increases cell death and reduces cell proliferation [34]. We did however observe a significant increase in cell viability (% of uninfected control) of A2780/cp70 cells infected with dl24 oncolytic Ad in the absence of cisplatin after HDAC2 knockdown by siRNA (siRNA1). There was no significant increase in cell viability in the presence of cisplatin after HDAC2 knockdown (Figure 3E). The reasons for this observation are not clear and require further investigation.

### Pan-HDAC inhibition does not influence cell viability in the absence of Ad

**Figure 4:**
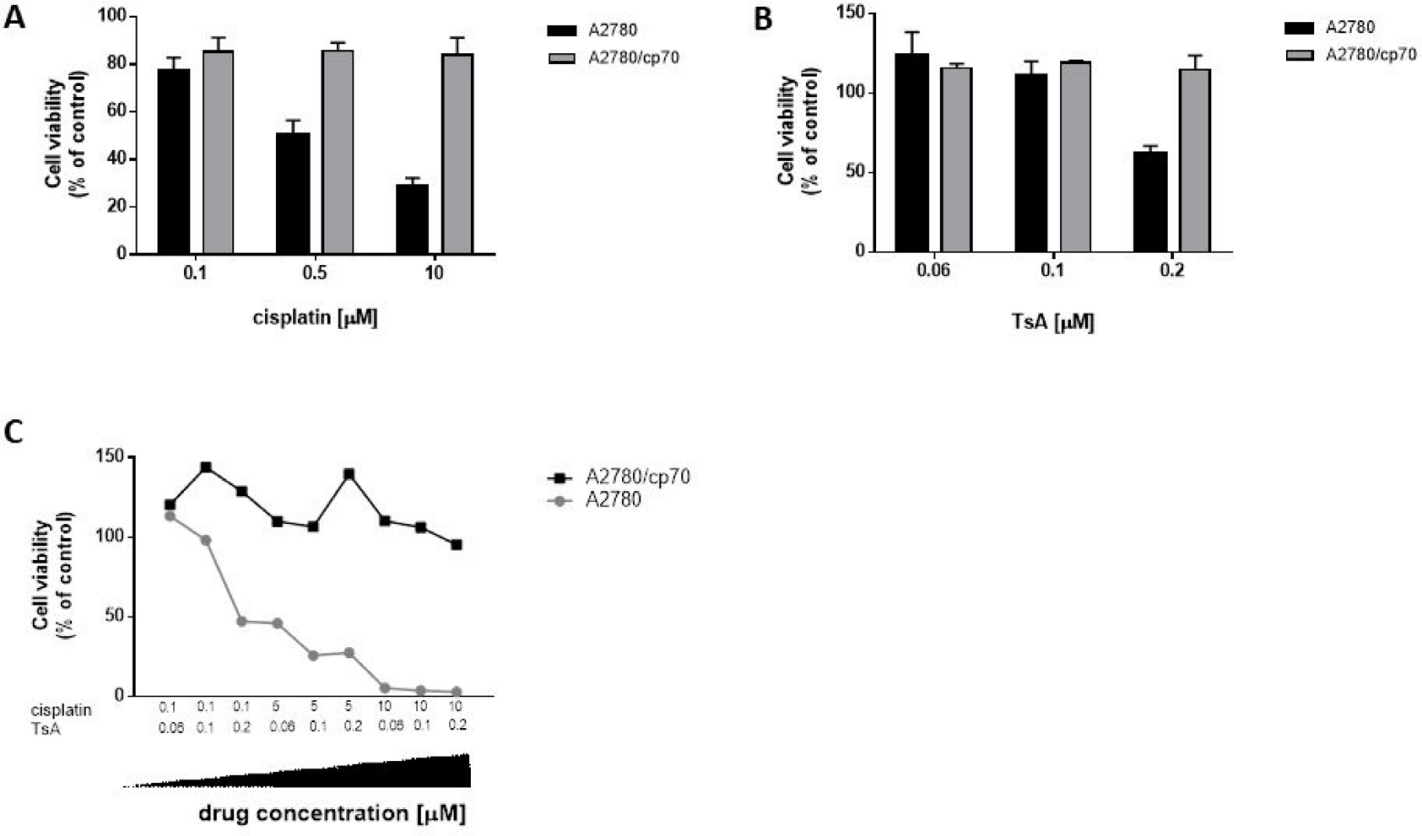
In the absence of Ad infection, A2780 (cisplatin-sensitive) cells show decreased cell viability with increasing doses of cisplatin, TsA and combination treatments whereas A2780/cp70 (cisplatin-resistant) cells are refractory to drug-induced reduction of cell viability (**A**) A2780 and A2780/cp70 cells were treated with cisplatin at either 0.1 µM, 0.5 µM or 10 µM cisplatin or (**B**) or TsA at 0.06 µM, 0.1 µM or 0.2 µM TsA, or (**C**) combinations of cisplatin and TsA. Cells were harvested at 72 h. Cell viability was measured by MTS assay and calculated as a percentage of untreated cells (no cisplatin or TsA). All values were corrected for background absorbance from the incubation media. Experiments were performed in triplicate. Data is expressed as the mean ± SEM.

To assess a potential role for HDAC over-expression on resistance to cancer therapies, we compared cell viability of cisplatin-sensitive and -resistant cells in the presence and absence of the pan-HDACi, trichostatin A (TsA). We applied cisplatin and TsA separately and in combination, measuring cell viability at 72 h to mimic the time-point for analysing cells post-infection with Ad (Figure 2). Cells were exposed to varying doses of cisplatin (0.1–10 µM, Figure 4A) and TsA (60 nM–0.2 µM, Figure 4B) and in combination (Figure 4C). A2780 cisplatin-sensitive cells were more sensitive to the combination cisplatin and TsA treatment, than to either drug alone, particularly at lower doses, whilst there was no meaningful impact on cytotoxicity of the combination in A2780/cp70 cisplatin-resistant cells (Figure 4C).

### TsA enhances Ad efficacy in cisplatin-resistant cells exposed to cisplatin

**Figure 5:**
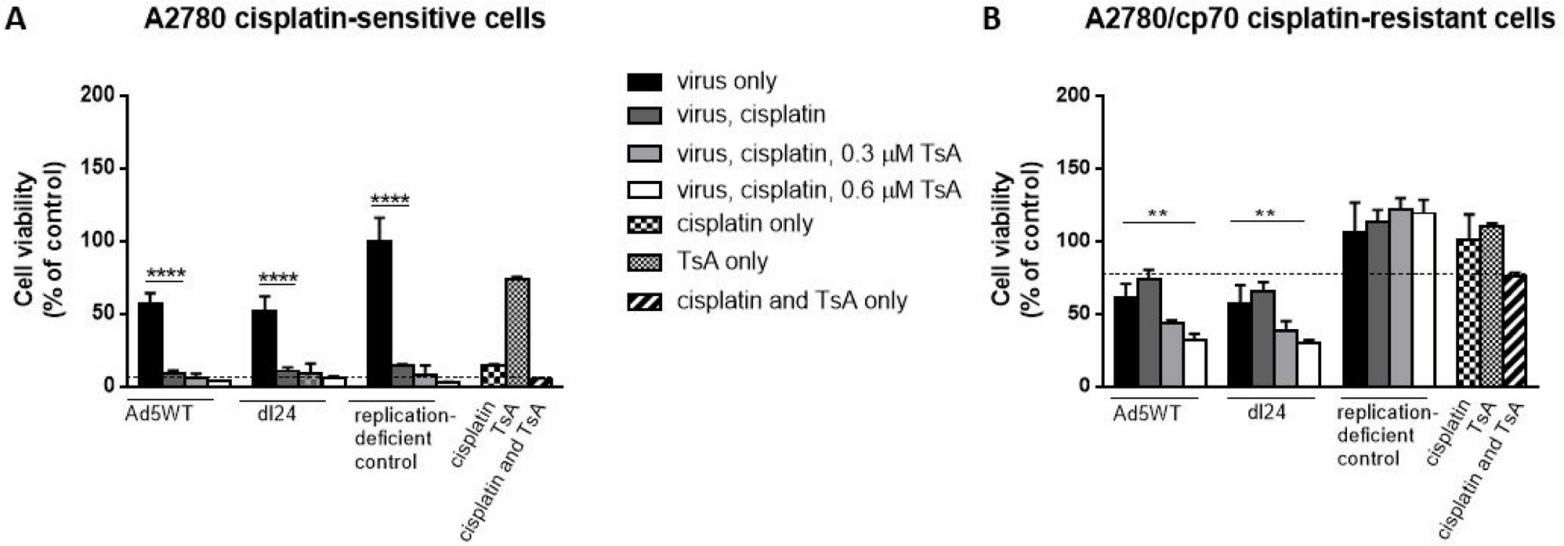
Cisplatin and TsA treatment sensitises A2780/cp70 cisplatin-resistant cells to Ad5WT (replication-competent) and dl24 (oncolytic) Ad (**A**) A2780 and (**B**) A2780/cp70 cell viability at 48 h post-infection (10 MOI) in the absence and presence of 20 µM cisplatin, 0.3 µM and 0.6 µM TsA and combination treatments. Cell viability was calculated as a percentage of uninfected cells (no Ad). Data was corrected for background absorbance from the incubation media. Experiments were performed in triplicate. Data is expressed as the mean ± SEM. Statistics were performed using the Dunnett`s multiple comparison test. *P* values ≤ 0.05 are considered statistically significant and calculated to determine differences between Ad infected cells and Ad infected cells in the presence of cisplatin.

We next tested whether histone modification in cisplatin-resistant cells influences Ad-mediated reduction of cell viability in the presence of cisplatin. Cells were infected with Ad (MOI = 10) and cisplatin (20 µM) without TsA, or in combination with either 0.3 µM or 0.6 µM TsA and cell viability measured at 48 h post-infection, time-points that allow Ad replication to occur (Figure 5). Cisplatin-sensitive A2780 cells infected with Ad5WT or dl24 resulted in ∼50% cell viability that decreased to ∼10% in combination with cisplatin treatment, but no further reduction in cell viability was achieved with TsA treatment (Figure 5A). Replication-deficient control Ad infection with cisplatin and TsA treatment alone or in combination was cytotoxic achieving similar levels of reduced cell viability as that observed with Ad5WT and dl24 oncolytic Ad infection. Cisplatin-resistant A2780/cp70 cells infected with either Ad resulted in ∼60% cell viability, increasing to ∼70% cell viability as expected with a chemo-resistant phenotype upon cisplatin treatment (20 µM) (Figure 5B). The development of chemo-resistance is likely due to multiple mechanisms including alterations in drug transport and increased efflux out of the cell, increased tolerance to drug-induced DNA damaging agents, altered drug target and DNA repair mechanisms [35]. Interestingly, treatment of Ad5WT infected, cisplatin treated cells with 0.3 µM TsA reduced cell viability from ∼70% to 43% cell viability), with a further reduction in cell viability to 31% with 0.6 µM TsA. Infection of cisplatin treated cells with dl24 and 0.3 µM TsA treatment reduced cell viability from ∼70% to 38%, decreasing further to 29% cell viability with 0.6 µM TsA treatment (*P* < 0.01). The non-replicating control Ad was not affected by cisplatin and TsA treatment indicating cisplatin-resistant cells require replicating Ad. The level of reduced cell viability is considered to be Ad-dependent as in the absence of infection of cisplatin-resistant A2780/cp70 cells, cisplatin, TsA and combination treatments had no effect on cell viability (94%, 110% and 87% cell viability respectively). Collectively, these findings show that pan-HDAC inhibition by TsA sensitises cisplatin-resistant (but not cisplatin-sensitive) cells to Ad.

### TsA sensitises cisplatin-resistant cells to Ad

**Figure 6:**
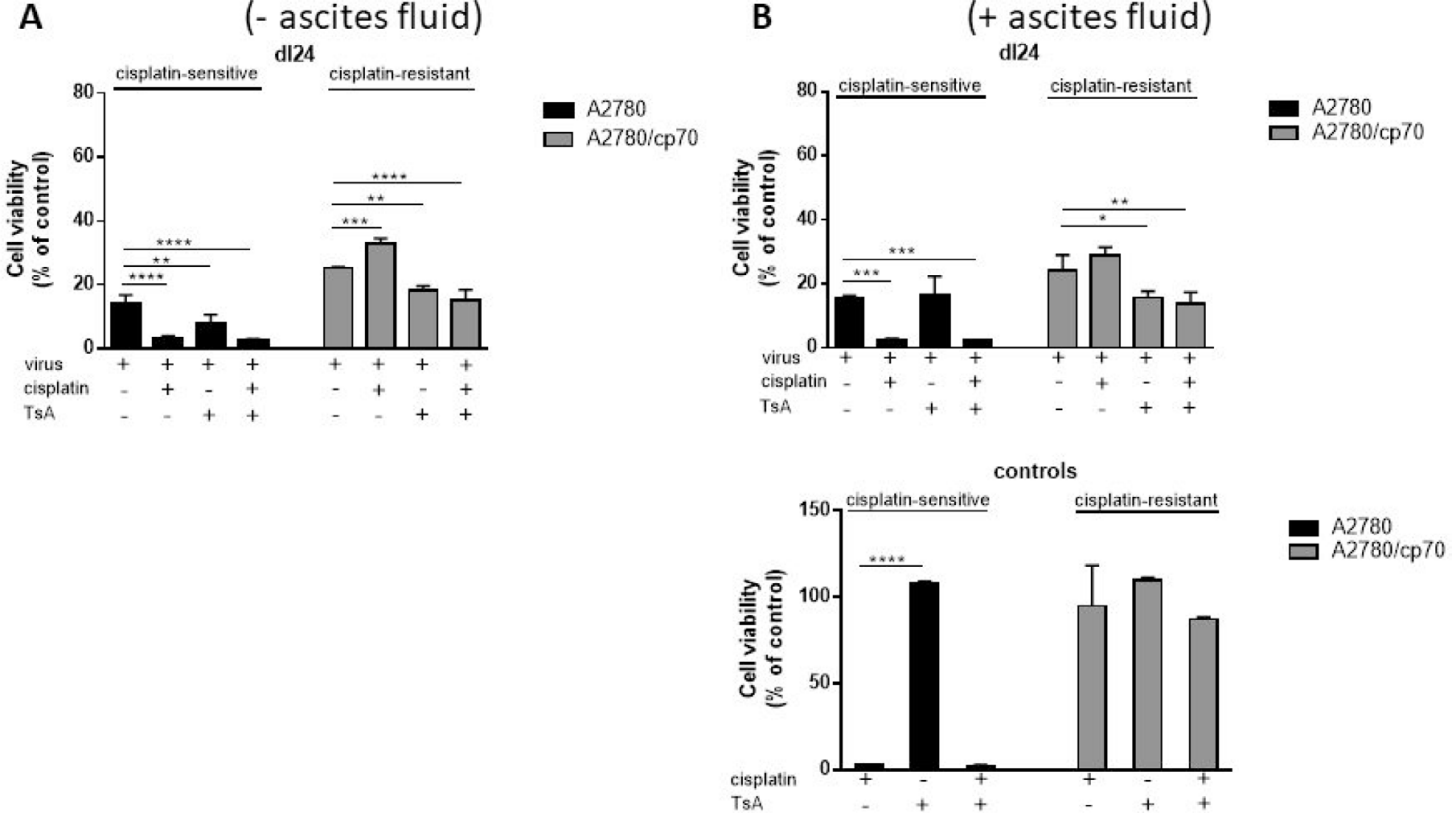
TsA and cisplatin/TsA are equally efficient at sensitising cisplatin-resistant cells to Ad, an effect not observed in cisplatin-sensitive cells where TsA has no effect Cell viability as measured at 72 h post-infection for each Ad infected cell (Ad5WT or dl24 at an MOI of 10) in the absence and presence of 20 µM cisplatin, 0.6 µM TsA and combination treatments was calculated as a percentage of uninfected cells. (**A**) Experiments performed without clinical ascites fluid in the culture media and (**B**) in the presence of clinical ascites fluid. Data was corrected for background absorbance from the incubation media. Experiments were performed in triplicate. Data is expressed as the mean ± SEM from 3 independent experiments. Statistics were performed using the Dunnett`s multiple comparison test. *P* values ≤ 0.05 are considered statistically significant and calculated to determine differences between Ad infected cells and Ad infected cells in the presence of cisplatin and or TsA.

We sought to determine the individual and combined effects of TsA and dl24 oncolytic Ad cytotoxicity at 72 h post-infection in cisplatin-sensitive and -resistant cells in the absence and presence of ascitic fluid from patient donors (Figure 6A). TsA (0.6 µM) had no significant impact upon the cytotoxicity achieved with OAd (MOI = 10) in cisplatin-sensitive cells, either alone or in combination with 20 µM cisplatin. In contrast, TsA produced a statistically significant increase in cytotoxicity achieved by OAd in cisplatin-resistant cells, both alone and with cisplatin. These findings support the suggestion, from Figure 5 above, that TsA selectively sensitises cisplatin-resistant cells to Ads but not to cisplatin.

We have previously shown that the cytotoxic effects of Ad5-based vectors are inhibited by the presence of pre-existing nAbs in ascitic fluid. In the presence of 2.5% ascitic fluid, levels of transduction with Ad5 were reduced by 98.9% [36]. To test whether the sensitising effect of TsA is affected by the presence of ascitic fluid, we performed parallel experiments to those in Figure 6A, but in the presence of 2.5% ascitic fluid (Figure 6B). Ascitic fluid seemed to have no meaningful quantitative or qualitiative impact on cytotoxicity due to the selectively replicating oncolytic Ad dl24, in either cisplatin-sensitive or -resistant cells, with or without cisplatin. Collectively, our findings show that TsA treatment selectively sensitises cisplatin-resistant cells to Ad but not to cisplatin.

### TsA does not alter CAR expression

To confirm whether TsA treatment of cells affects the levels of CAR expression, cells were analysed by flow cytometry 24 hours after culture in the presence or absence of 0.6 µM TsA (data not shown). Cultured A2780 cisplatin-sensitive cells demonstrated low levels of CAR expression. A2780/cp70 cisplatin-resistant cells showed two populations of cells that were negative and positive (22.4%) for CAR expression. TsA treatment slightly increased CAR expression in the sub-population of CAR negative cells, however this was not statistically significant.

## DISCUSSION

The results of clinical trials testing OAds for targeted cancer applications have thus far been rather disappointing [4, 37–40]. In this study, we tested the ability of Ad5WT and dl24 oncolytic Ad to reduce cell viability in cisplatin-sensitive and -resistant ovarian cancer cells. We show that both Ads reduce cell viability in cisplatin-sensitive cells. However, we observed significantly increased cell viability for cisplatin-resistant cells infected with Ad5WT and dl24 oncolytic Ad and escalating dose of cisplatin. We hypothesised that the observed reduction in Ad efficacy with increasing cisplatin may be due to chromatin modifications (HDACs) in cisplatin-resistant cells that affect Ad replication, although elucidating the mechanisms for these observations are not within the scope of this study.

Previous studies consider HDAC1, –3 and –7 as tumour biomarkers [41] and knockdown of HDAC1, –2, –3 and –6 in a variety of cancer cell lines can promote apoptosis and cell cycle arrest while HDAC1, –2 and -4 are required to maintain cancer cell survival *in vivo* [42]. Indeed Jones [43], describes the use of epigenetic therapies as an attractive strategy for treating solid cancers due to the ability of HDACis to cause global alterations in the epigenome, however the author suggests the use of HDACis alone are unlikely to be efficacious unless combined with other modalities such as chemotherapy. An interesting study reported enhanced efficacy of a combination of TsA and 5-aza-2`-deoxycitidine (DNA-methyltransferase inhibitor) and low-dose cisplatin in comparison to single drug *in vitro* as measured by decreased ovarian cancer cell line cell viability, migration and spheroid growth [44]. Furthermore, administration of cisplatin followed by TsA significantly suppressed tumourigenicity in a mouse xenograft model. In the context of virotherapy, we hypothesised that differences in HDAC expression in chemo-resistant cells may hinder the ability of Ads to utilise the host`s cellular proteins for efficient cell entry, replication and/or promote chemo-resistance by preventing nuclear access of cisplatin. In support of this idea, we found that basal HDAC2 expression (and to a lesser extent HDAC1) is up-regulated in cisplatin-resistant cells. This has also been demonstrated in PE01 (cisplatin-sensitive) and PE04 (cisplatin-resistant) ovarian cancer cells soon after cisplatin treatment (24 h), the authors suggesting changes in nuclear texture by HDAC2 are possibly a mediator of an early DNA damage response in sensitive tumour cells [45]. Histone deacetylation is a form of self-defence to injury thereby repressing transcription, initiating chemotherapy-triggered DNA damage repair and promoting survival followed by histone acetylation to produce open chromatin for DNA accessibility downstream of the injury sites. In the present study, knockdown of HDAC2 failed to significantly reduce cell viability of A2780/cp70 cisplatin-resistant cells. Previous studies have shown decreased cell viability of the parental A2780 cells after HDAC2 knockdown [46], whilst others have reported no reduction in cell viability without simultaneous knockdown of HDAC1 [47, 48]. Previous studies have shown that HDAC2 depletion by siRNA reduced the IC_50_ of cisplatin in PE01 cells suggesting loss of HDAC2 enhances the effect of cisplatin [45], however this observation in response to cisplatin may be a separate mechanism to that of cell viability in response to adenovirus infection as shown by the findings of our study.

A limitation of our study is that other HDACs are likely to be involved in maintaining the phenotype of cisplatin-resistant cells, in particular HDAC1 which exhibits very close sequence homology and co-exists with HDAC2. In support of this, we investigated whether pan-HDAC inhibition (by TsA treatment) might enhance Ad efficacy in cisplatin-resistant cells. TsA alone did not affect viability of A2780 or A2780/cp70 cells. Cisplatin-sensitive A2780 cells infected with Ad and TsA demonstrated no difference in cell viability, but addition of cisplatin reduced cell viability, again confirming a cisplatin-mediated effect. Interestingly, the opposite effect was observed for cisplatin-resistant cells where TsA treatment significantly enhanced Ad-mediated reduction of cell viability, even in the presence of high dose cisplatin, suggesting that TsA sensitises cisplatin-resistant cells to Ad but not cisplatin. Although the addition of 600 nM TsA to Ad infected A2780/cp70 cisplatin-resistant cells decreased cell viability, 30% of cells were still remaining, which would not be a satisfactory outcome clinically. However, in this study, we only tested TsA at 300 nM and 600 nM doses as described previously [44, 49] and showed a decrease in cell viability with increasing dose of TsA. It is possible that increasing the dose of TsA further, may sensitise cisplatin-resistant cells to Ad to a level sufficient for reaching clinical efficacy.

Earlier studies show enhanced anti-tumour effects of Ad co-treated with the HDACi FR901228 in lung cancer cells [50] and FK228 (romidepsin) administered prior to Ad infection boosted infection in a melanoma xenograft model [51]. HDAC inhibition has been shown to up-regulate CAR expression in bladder cancer [52], although our data did not show an increase in CAR expression with TsA treatment suggesting TsA acts to enhance Ad efficacy intra-cellularly. The OAd Delta24-RGD previously shown to lack infectivity of glioma cells demonstrated enhanced Ad infection through up-regulated α_v_β3 integrin when combined with Scriptaid and LBH589 through up-regulation of multiple cell death pathways [53]. A combination of Ad E1A gene therapy and SAHA (suberanilohydroxamic acid or Vorinostat) showed high therapeutic efficacy and low toxicity using *in vivo* ovarian and breast xenograft models [54].

Consistent with the potential for developing Ads for intra-peritoneal delivery in ovarian cancer patients, we repeated our experiments in the presence of *ex vivo* ascitic fluid. The accumulation of ascites represents a hallmark feature of ovarian cancer and contains pre-existing nAbs to Ad5 that may preclude the use of un-modified Ad5-based vectors. We have previously reported that in the presence of 2.5% ascitic fluid (patient OAS001), levels of Ad transduction in EOC cells were reduced by 98.9% [36]. Our findings show dl24 Ad enhanced cisplatin-resistant reduction in cell viability with TsA treatment, even in the presence of ascitic fluid.

In conclusion, this study demonstrates that Ad-mediated reduction of cell viability is decreased with increasing doses of cisplatin in cisplatin-resistant ovarian cancer cells. Pan-HDAC inhibition by TsA treatment sensitises cisplatin-resistant cells to Ad, even in the presence of high dose cisplatin and *ex vivo* ascitic fluid. These are novel findings with potential clinical implications for the use of Ad vectors in chemo-resistant end-stage disease.

## MATERIALS AND METHODS

### Materials

Cisplatin was obtained from the Velindre Cancer Centre, Cardiff. Trichostatin A (TsA) was purchased from Sigma-Aldrich. Both reagents were suspended in complete incubation media supplemented with 2% fetal calf serum. The CellTiter 96^®^ AQ_ueous_ One Solution Cell Proliferation Assay [3-(4,5-dimethylthiazol-2-yl)-5-(3-carboxymethoxyphenyl)-2-(4-sulfophenyl)-2H-tetrazolium)] (MTS) was purchased from Promega. HDAC antibodies (rabbit monoclonal) against human HDAC1, -2 and -3 antibodies were purchased from Abcam (ab109411, ab32117, ab32369 respectively). HDAC2 siRNAs were purchased from Qiagen.

### Ethics approval

Ethics permission for the collection of ascites was granted through a Wales Cancer Bank application for biomaterials, reference WCB 14/004. All patients gave written informed consent for the use of their samples, prior to collection.

### Generation of Ad vectors

Ad5WT was generated by AdZ homologous recombineering as previously described [55]. dl24 was generated from Ad5WT by AdZ homologous recombineering and rendered conditionally replicative (oncolytic) by deletion of 24 base pairs in the E1A region involved in binding Rb protein as previously described [33]. Hence dl24 is unable to induce S phase for Ad replication, restricting replication to actively dividing cells such as in the tumour microenvironment. All Ads were diluted in complete incubation media supplemented with 2% fetal calf serum.

### Primary epithelial ovarian cancer (EOC) cells

Ascites samples were collected from patients with ovarian cancer at the Velindre Cancer Centre, Cardiff, UK and anonymously coded (EOC003 and EOC009). Ascites was stored at 4° C immediately after collection and processed within 24 h. Approximately 400 mL of ascites was centrifuged at 1000 rpm for 5 min to separate primary EOC cells from the fluid. The supernatant was stored at –70° C for subsequent use with autologous tumour cells. Red blood cell lysis buffer (Sigma Aldrich, UK) was added to the pellet according to the manufacturer`s instructions, where appropriate. Tumour cell pellets were frozen in 10% DMSO and 90% autologous supernatant (passage 0). A further 100 mL of ascites was used to generate primary EOC cultures, by separating into 20 mL aliquots and adding to 20 mL of complete (RPMI 1640) medium, supplemented with 10% (v/v) fetal calf serum (FCS), 200 µM glutamine, 100 U/mL penicillin, 100 µg/mL streptomycin and 10% (v/v) autologous ascitic fluid supernatant. Cells were maintained at 37° C and 5% CO_2_. The resulting primary cultures were passaged when cells had reached confluence. All reagents were purchased from Gibco or Thermo Scientific (Paisley, UK).

### SKOV3, A2780 and A2780/cp70 cell lines

SKOV3 cells (human ovarian adenocarcinoma derived from ascites) were originally obtained from the American Type Culture Collection (ATCC). A2780 parental (cisplatin-sensitive) human ovarian carcinoma and A2780/cp70 (cisplatin-resistant) human ovarian carcinoma cells were a kind gift from Dr Alwyn Dart, Cardiff University. Cells were cultured as described above and passed a mycoplasma test in November 2016.

### Coxsackie adenovirus receptor (CAR) expression

EOC, SKOV3, A2780 and A2780/cp70 cells were seeded at a density of 1.5 × 10^5^ cells per well in a 96-well plate. Cells were washed in 200 µl of wash buffer (PBS/1% BSA) and incubated with 100 µl of wash buffer containing 1:500 of mouse anti-human monoclonal antibody against CAR (RmcB, Millipore, Watford, UK) or mouse IgG control antibody (Santa Cruz Biotechnology, Heidelberg, Germany) for 1 hour on ice. Cells were washed three times and incubated with a 1:500 dilution of goat anti-mouse Alexa Fluor 647 antibody (Invitrogen, UK) for 30 min on ice. Cells were fixed in 4% paraformaldehyde for 20 min at 4° C. 2 × 10^4^ gated events were acquired in channel FL-4 on a BD Accuri C6 (BD Biosciences, USA) flow cytometer and data analysed in BD Accuri C6 software version 1.0.264.21 (Becton Dickinson, USA). hCAR expression was analysed by flow cytometry relative to IgG isotype expression.

### Ad infection and cell viability

Cells were seeded in 96-well plates at a density of 2 × 10^4^ cells/well in 100 µl of RPMI media supplemented with 10% fetal calf serum, 2 mM L-glutamine, 100 U/ml penicillin and 100 µg/ml streptomycin. Cells were incubated in humidified boxes at 5% CO_2_, 37° C. After 24 h, cells were infected with Ad at an MOI of 0–10 (calculated as the ratio between the number of Ad particles and number of cells) and cultured as described above. Cell viability (% relative to uninfected cells) was measured by MTS assay. In brief, 20 µl of CellTiter 96^®^ AQ_ueous_ One Solution Cell Proliferation Assay reagent was added to 100 µl of culture medium, followed by incubation at 5% CO_2_, 37° C for 2 h. Plates were read at 490 nM using a multimode plate reader (FLUOstar Omega, BMG Labtech, Aylesbury, UK). Cell viability for cells infected with Ad was calculated as a percentage of uninfected cells (control). All values were corrected for background absorbance from the incubation media. All experiments were performed in triplicate. Data are expressed as the mean ± SEM.

### Ad infection in combination with cisplatin dose escalation and cell viability

Cells were seeded in 96-well plates at a density of 2 × 10^4^ cells/well in RPMI media supplemented with 10% fetal calf serum, 2 mM L-glutamine, 100 U/ml penicillin and 100 µg/ml streptomycin. Cells were incubated in humidified chambers at 37° C, 5% CO_2_. After 24 h, cells were infected with Ad at 100 MOI in RPMI media supplemented with 2% fetal calf serum, 2 mM L-glutamine, 100 U/ml penicillin and 100 µg/ml streptomycin. At 2 h post-infection, cisplatin was added to cells at doses of 0.01, 1, 10 and 20 µM. Cisplatin only cells were treated with 20 µM cisplatin. Cell viability was measured at 72 h post-infection by MTS assay as described above. Cell viability for each Ad infected cell in the absence and presence of cisplatin was calculated as a percentage of uninfected cells (no Ad or cisplatin). Data was corrected for background absorbance from the incubation media. Experiments were performed in triplicate. Data are expressed as the mean ± SEM from 3 independent experiments. Statistics were performed using the Dunnett`s multiple comparison test. *P* values ≤ 0.05 are considered statistically significant and calculated to determine differences between adenovirus infected cells and adenovirus infected cells in the presence of cisplatin.

### Ad transduction

Assays were performed as previously described [13]. In brief, cells were seeded at a density of 2 × 10^4^ cells/well in a 96-well plate. After 24 hours, cells were infected with a luciferase expressing dl24 Ad at doses of 1000, 5000 and 10 000 virus particles (vp) per cell in a total volume of 100 µl of serum-free medium and incubated at 5% CO_2_, 37° C for 3 hours. The medium was removed and replaced with 200 μl of complete medium (RPMI 1640 medium supplemented with 10% (v/v) fetal calf serum, 100 U/mL penicillin, 100 µg/mL streptomycin) and cultured for an additional 45 hours. Cells were lysed in 1 × Cell Culture Lysis Buffer (Promega, UK) and frozen at –70° C. The cells were thawed and 20 µl of cells mixed with 100 µl of luciferase assay reagent in a white 96-well plate. Luciferase activity in relative light units (RLU) was measured immediately using a multimode plate reader (FLUOstar Omega, BMG Labtech, Aylesbury, UK). Samples were normalised for total protein content, as measured by bicinchoninic acid (BCA) assay in RLU/mg protein. 2 × 10^4^ gated events were acquired in channel FL-1 on a BD Accuri C6 using the plate reader as described above.

### Intracellular HDAC expression

A2780 and A2780cp/70 cells were seeded at a density of 1 × 10^5^ cells per well, fixed with 4% paraformaldehyde, permeabilised with 0.1% Triton-X100 in PBS and blocked with 10% normal goat serum. Cells were incubated with rabbit anti-HDAC1, anti-HDAC2 and anti-HDAC3 antibodies (1:50) (Abcam, Cambridge, UK) and detected with Alexa Fluor 488 goat anti-rabbit secondary antibody (1:2000) (Invitrogen, Paisley, UK). 2 × 10^4^ gated events were acquired in channel FL-1 on a BD Accuri C6 using the plate reader as described above. HDAC expression was determined relative to the IgG isotype expression.

### HDAC2 siRNA transfection

A2780/cp70 (cisplatin-resistant cells) were seeded at 10 × 10^4^ cells per well in a 96-well plate in complete incubation media in the absence of antibiotics. 24 h after seeding, cells were transfected with 6 pmol HDAC2 siRNAs, All Stars negative control (scrambled) or AbI 1 5 (positive control) (Qiagen) and 0.3 µl Lipofectamine reagent per well in OptiMEM media (in triplicate). Transfection control wells contained nuclease-free water and OptiMEM media only. Cells were incubated in a humidified box at 37° C, 5% CO_2._ For cisplatin wells, cells were incubated for 2 h then dosed with 20 µM cisplatin. Cell viability was measured at 48 h by MTS assay and calculated as a percentage of non-transfected cells. All values were corrected for background absorbance from the incubation media. Experiments were performed in triplicate. Data is expressed as the mean ± SEM.

### Western blotting

Cells were lysed in 1% SDS lysis buffer (50 mM Tris HCl, pH 6.8, 10% glycerol, 1% SDS). Each triplicate wells were pooled. 10 µl of each sample were used for a BCA assay. Samples were freeze thawed and diluted as appropriate with water to 10 µg. Samples were centrifuged at full speed and the same volume of 2 X Laemmli buffer (4% SDS, 20% glycerol, 10% 2-mercaptoethanol, 0.004% bromophenol blue, 0.125M Tris HCl) added to each sample. Samples were boiled at 95° C for 10 mins. Samples were loaded onto a 10% pre-cast Bis-Tris SDS-PAGE gel and electrophoresed at 200 V for 1 h. Gels were transferred at 20 V for 1 h (semi-dry transfer) to a nitrocellulose membrane. Membranes were blocked in 1 X TBST, 2% BSA at 37° C, room temperature (RT) 1 h. The siRNA membrane was incubated with rabbit anti-HDAC2 antibody (ab32117) (1:2000) in 1 X TBST, 2% BSA and the positive control siRNA AbI 1 membrane was incubated in mouse anti-ABI antibody (1:200) in 1 X TBST, 2% BSA overnight at 4° C. Membranes were washed 3 × 10 min in 1 X TBST. Anti-rabbit HRP and anti-mouse HRP (1:2000) in 1 X TBST, 2% BSA was added to each membrane respectively and incubated at RT for 1 h. Membranes were washed 3 × 10 min in 1 X TBST. Membranes were incubated with West Pico chemiluminescent reagent and immediately imaged. For actin loading control, membranes were stripped (Restore Western Blot Stripping buffer) for 15 min at RT and washed with 1 X TBST and incubated with rabbit anti-actin antibody (1:2000) in 1 X TBST, 2% BSA for 1 h at RT. Membranes were washed 3 × 10 min in 1X TBST. Membranes were incubated with anti-rabbit HRP antibody (1:2000) in 1X TBST, 2% BSA for 1 h at RT. Membranes were washed 3 × in 1 X TBST and incubated with West Pico. Membranes were imaged immediately.

### Cisplatin and TsA dose response in the absence of Ad and cell viability

A2780 and A2780/cp70 cells were seeded at 2 × 10^4^ cells/well in RPMI media supplemented with 10% fetal calf serum, 2 mM L-glutamine, 100 U/ml penicillin and 100 µg/ml streptomycin in 96-well plates incubated in humidified boxes at 37° C, 5% CO_2_. Cells were treated with cisplatin at either 100, 50, 10 or 1 µM and TsA at either 2 µM, 1 µM or 600 nM as described above. Combinations of each dose of cisplatin and TsA were also tested. Cells were harvested at 72 h. Cell viability was measured by MTS assay (as described above) and calculated as a percentage of untreated cells (no cisplatin or TsA). All values were corrected for background absorbance from the incubation media. Experiments were performed in triplicate. Data are expressed as the mean ± SEM.

### Efficacy of Ad in combination with cisplatin and TsA co-treatment

A2780 and A2780cp/70 cells were seeded at 2 × 10^4^ cells/well in complete media containing 10% fetal calf serum, 2 mM L-glutamine, 100 U/ml penicillin and 100 µg/ml streptomycin in 96-well plates incubated in humidified boxes at 37° C, 5% CO_2_. After 24 h, cells were infected with Ad5WT, dl24 or control Ad at 100 MOI in RPMI media supplemented with 2% fetal calf serum, 2 mM L-glutamine, 100 U/ml penicillin and 100 µg/ml streptomycin. At 2 h post-infection, 20 µM cisplatin and either 300 nM or 600 nM TsA [36] was added to cells. Cisplatin only wells were treated with 20 µM cisplatin. TsA only cells were treated with 600 nM TsA. Cells were harvested at 24 h, 48 h, 72 h and 144 h. Cell viability was measured by MTS assay as described above. Cell viability for each Ad infected cell in the absence and presence of cisplatin and or TsA was calculated as a percentage of uninfected cells (no Ad). All values were corrected for background absorbance from the incubation media. Experiments were performed in triplicate. Data are expressed as the mean ± SEM. *P* values ≤ 0.05 were considered statistically significant and calculated to determine differences in cell viability between Ad infected cells in the presence of cisplatin and adenovirus infected cells in the presence of cisplatin and TsA.

### Efficacy of Ad in combination with cisplatin and TsA co-treatment in the presence of clinical *ex vivo* ovarian cancer ascitic fluid

A2780 and A2780cp/70 cells were seeded in 96-well plates at a density of 2 × 10^4^ cells / well in RPMI media supplemented with 10% fetal calf serum, 2 mM L-glutamine, 100 U/ml penicillin and 100 µg/ml streptomycin. Cells were incubated in humidified boxes at 37° C, 5% CO_2_. After 24 h, cells were infected with Ad5WT or dl24 at 100 MOI in RPMI media supplemented with 2% fetal calf serum, 2 mM L-glutamine, 100 U/ml penicillin and 100 µg/ml streptomycin. At 2 h post-infection, 20 µM cisplatin and/or 600 nM of TsA and 2.5% ascitic fluid containing highly neutralising antibodies to Ad5 (patient OAS001) [36] was added to cells. Cisplatin only wells were treated with 20 µM cisplatin. TsA only cells were treated with 600 nM TsA. Cells were harvested at 24 h, 48 h and 72 h. Cell viability was measured by MTS assay as described above. Cell viability for each adenovirus infected cell in the absence and presence of cisplatin and or TsA was calculated as a percentage of uninfected cells (no Ad). All values were corrected for background absorbance from the incubation media. Experiments were performed in triplicate. Data are expressed as the mean ± SEM. The broken line indicates the percentage of cell viability of non-infected cells incubated with cisplatin and TsA only.

### TsA treatment and CAR expression

Cells were seeded at a density of 2 × 10^4^ cells per well (day 0). After 24 h (day 1), cells were treated with TsA (600 nM). Cells were maintained at 37° C, 5% CO_2_ in a humidified chamber. At 24 h post-treatment, cells were stained for CAR expression. Primary antibodies were mouse IgG isotype control antibody (Santa Cruz Biotechnology, Heidelberg, Germany) (1:200) or mouse anti-hCAR antibody (RmcB, Millipore, Watford, UK) (1:500) for 1 h at 4° C followed by detection with anti-mouse Alexa Fluor 647 (Invitrogen, UK) (1:500) for 45 min at 4° C. Cells were fixed in 4% paraformaldehyde for 20 min at 4° C. CAR expression was analysed by flow cytometry relative to IgG isotype expression using the BD Accuri C6 as described above.

### Statistical analyses

Data are presented as experiments performed in triplicate. Analysis of variance (ANOVA) and the Dunnett’s or Sidak`s multiple comparisons post hoc test was performed when three or more groups of data were analysed. All analyses and graphs were created in GraphPad Prism version 6.03 (GraphPad Software Inc., La Jolla, CA, USA).

## SUPPLEMENTARY MATERIALS FIGURE



## Abbreviations

EOC: epithelial ovarian cancer
OAd: oncolytic adenovirus)
Ad: adenovirus
Ad5: adenovirus serotype 5
Ad5WT: adenovirus serotype 5 wild-type)
dl24: Ad5 serotype 5 ∆24
CAR: coxsackie adenovirus receptor
HDAC: histone deacetylase
HDACi: histone deacetylase inhibitor
HDACis: histone deacetylase inhibitors
TsA: trichostatin A
nAbs: neutralising antibodies
GM-CSF: granulocyte-macrophage colony-stimulating factor
HSV-1: herpes simplex virus
