# Supplementary material for: Histone deacetylase inhibitor trichostatin A sensitises cisplatin-resistant ovarian cancer cells to oncolytic adenovirus
Source: Oncotarget. 2018 May 29;9(41):26328–41. doi: 10.18632/oncotarget.25242 (PMC5995174; doi:10.18632/oncotarget.25242)
Supplement: Supplementary file 1 [file oncotarget-09-26328-s001.pdf]

Histone deacetylase inhibitor trichostatin A sensitises cisplatin-resistant ovarian cancer cells to oncolytic adenovirus

SUPPLEMENTARY MATERIALS

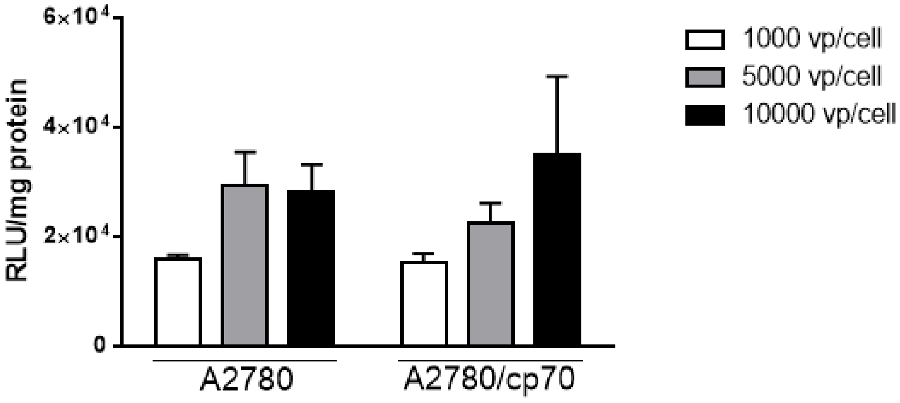

Supplementary Figure 1: dl24 transduction is similar in A2780 and A2780/cp70 cells. Cells were infected with dl24 (luciferase expressing Ad) at 1000 vp/cell, 5000 vp/cell and 10,000 vp/cell. Cell transduction was measured by luciferase activity at 48 h post-infection and normalised for protein content by bicinchoninic acid assay. Experiments were performed in triplicate. Data are expressed as the mean ± SEM.
